# Analysis of Homozygous-by-Descent (HBD) Segments for Purebred and Crossbred Pigs in Russia

**DOI:** 10.3390/life11080861

**Published:** 2021-08-22

**Authors:** Siroj Bakoev, Anatoly Kolosov, Faridun Bakoev, Olga Kostyunina, Nekruz Bakoev, Timofey Romanets, Olga Koshkina, Lyubov Getmantseva

**Affiliations:** 1 Don State Agrarian University, Persianovski 346493, Russia; siroj1@yandex.ru (S.B.); kolosov777@gmail.com (A.K.); bakoevfaridun@yandex.ru (F.B.); timofey9258@mail.ru (T.R.); 2 Centre for Strategic Planning and Management of Biomedical Health Risksdisabled, Moscow 119435, Russia; 3 Federal Research Center for Animal Husbandry named after Academy Member LK. Ernst, Dubrovitsy 142132, Russia; kostolan@yandex.ru (O.K.); nekruz82@bk.ru (N.B.); olechka1808@list.ru (O.K.)

**Keywords:** homozygous-by-descent (HBD), autozygosity, inbreeding coefficient, deleterious variation, pig

## Abstract

Intensive selection raises the efficiency of pig farming considerably, but it also promotes the accumulation of homozygosity, which can lead to an increase in inbreeding and the accumulation of deleterious variation. The analysis of segments homozygous-by-descent (HBD) and non-HBD segments in purebred and crossbred pigs is of great interest. Research was carried out on 657 pigs, of which there were Large White (LW, *n* = 280), Landrace (LR, *n* = 218) and F1 female (♂LR × ♀LW) (F1, *n* = 159). Genotyping was performed using the GeneSeek^®^ GGP Porcine HD Genomic Profiler v1 (Illumina Inc., USA). To identify HBD segments and estimate autozygosity (inbreeding coefficient), we used the multiple HBD classes model. LW pigs exhibited 50,420 HBD segments, an average of 180 per animal; LR pigs exhibited 33,586 HBD segments, an average of 154 per animal; F1 pigs exhibited 21,068 HBD segments, an average of 132 per animal. The longest HBD segments in LW were presented in SSC1, SSC13 and SSC15; in LR, in SSC1; and in F1, in SSC15. In these segments, 3898 SNPs localized in 1252 genes were identified. These areas overlap with 441 QTLs (SSC1—238 QTLs; SSC13—101 QTLs; and SSC15—102 QTLs), including 174 QTLs for meat and carcass traits (84 QTLs—fatness), 127 QTLs for reproduction traits (100 QTLs—litter traits), 101 for production traits (69 QTLs—growth and 30 QTLs—feed intake), 21 QTLs for exterior traits (9 QTLs—conformation) and 18 QTLs for health traits (13 QTLs—blood parameters). Thirty SNPs were missense variants. Whilst estimating the potential for deleterious variation, six SNPs localized in the NEDD4, SEC11C, DCP1A, CCT8, PKP4 and TENM3 genes were identified, which may show deleterious variation. A high frequency of potential deleterious variation was noted for LR in DCP1A, and for LW in TENM3 and PKP4. In all cases, the genotype frequencies in F1 were intermediate between LR and LW. The findings presented in our work show the promise of genome scanning for HBD as a strategy for studying population history, identifying genomic regions and genes associated with important economic traits, as well as deleterious variation.

## 1. Introduction

Improving the efficiency of livestock production is associated with intensive selection and different breeding strategies. High-intensity selection promotes genetic progress, but it can lead to an undesirable increase in the level of inbreeding in purebred livestock [1,2]. In creating breeds of farm animals, the accumulation of homozygosity is the main goal, which allows the purebred animals not only to possess certain qualities, but also to steadily pass them on to their offspring. Furthermore, the intensive selection of highly productive animals leads to an increase in the frequency of homozygotes for deleterious variation [3]. In fact, inbred depression is an unintended result of selection, which is based on the recessive load of the individual, since the harmful alleles are already embedded in the population but are present in a heterozygous state [4]. There are various approaches to inbreeding estimation [5,6]. However, the development of technologies for obtaining genome information opens up new possibilities for controlling the level of genomic inbreeding. Runs of Homozygosity (ROH) are a direct consequence of inbreeding. According to Bosse et al. [7], “Inbreeding is the inheritance of identical copies of genetic material from related parents and causes long homozygous regions in the genome of the offspring (ROH: Runs Of Homozygosity)”. ROH provide a more accurate prognosis and are widely used in human and animal research for the accurate estimation of the autozygosity level [5,8,9,10,11,12,13].

The history of a population can be quite complex, and common ancestors belong to different generations. This frequently occurs in small populations, or in populations under strong selection [14]. In this connection, Druet and Gautier [14] presented an approach to solving this problem on the basis of the HBD multiple class model (HBD—homozygous-by-descent). Unlike ROH, the sequence of HBD and non-HBD segments is modeled by means of the hidden Markov model (HMM). As a result, the total autozygosity can be divided according to the age of the inbreeding event. An advantage of analyzing HBD segments is that they can be reliably identified even in the presence of systematic errors in the set of markers, uneven distance between markers and variable rates of genotyping errors due to the relatively low density of markers [14]. The ability to assess autozygosity according to the ancestors‘ age, to calculate the individual autozygosity of each animal, and use genotyping based on a relatively low density of markers makes this method attractive as a possible tool for managing breeding programs in animal husbandry.

In many countries, including the Russian Federation, pig breeding is based on a three-level pyramidal structure. At the first level, Large White and Landrace breeds are used to obtain F1 hybrid sows. Next, F1 sows are mated with Duroc boars, and the result is F2 final hybrids. In this connection, the analysis of HBD and not HBD segments in purebred and hybrid livestock is of great interest.

The objective of the work was to analyze homozygous-by-descent (HBD) segments for purebred and crossbred pigs in Russia. The tasks of this work were as follows: (i) to determine the autozygosity throughout the genome in Large White pigs, Landrace and F1 hybrids; (ii) to assess the contribution of different HBD classes to autozygosity; (iii) to determine the level of inbreeding from the sum of all HBD classes; (iv) to identify and characterize HBD segments in purebred and hybrid pigs; (v) to identify long segments of HBD and examine them for the presence of QTLs, genes and possible deleterious variations.

## 2. Materials and Methods

### 2.1. Animals

Anesthesia, euthanasia or any animal sacrifice was not used to conduct this study. This study does not involve any endangered or protected species. According to standard monitoring procedures and guidelines, the participating holding specialists collected tissue samples, following the ethical protocols outlined in the Directive 2010/63/EU (2010). The pig ear samples (ear pluck) were obtained under a general breeding monitoring procedure. The collection of ear samples is a standard practice in pig breeding [15].

Research was carried out on 657 pigs, of which there were Large White (LW, *n* = 280), Landrace (LR, *n* = 218) and F1 female (♂LR × ♀LW) (F1, *n* = 159). Genomic DNA was extracted from ear samples using a DNA-Extran-2 reagent kit (OOO NPF Sintol, Moscow, Russia) following the manufacturer’s protocol. The quantity and quality of DNA were assessed using a Qubit 2.0 fluorometer (Invitrogen/Life Technologies, Waltham, MA, USA) and a NanoDrop8000 spectrophotometer (ThermoFisher Scientific, Waltham, MA, USA).

### 2.2. Data Processing and Data Analyses

Genotyping was performed by using the GeneSeek^®^ GGP Porcine HD Genomic Profiler v1 (Illumina Inc., San Diego, CA, USA). Genotype quality control was performed using PLINK 1.9. We limited ourselves to removing SNPs with a call rate below 95%. After QC, 62,331 SNPs remained.

A popular class of probabilistic models, the hidden Markov model (HMM), was used to identify HBD and non-HBD segments in the genome of an organism. An unobservable HBD status assessed at each marker position is considered a hidden state. In order to calculate the probability of transition from one state to another, the HMM requires the probability of continuing or ending the current segment and the probability of observing genotypes. The probability of genotype detection depends on the HBD status, allele frequency, genotyping error rate and the mutation rate. Using the model of hidden Markov chains and genotypes of an organism in the form of a sequence, it is necessary to find such a sequence of states that would best describe the genotypes in a given model. To calculate the probability of a certain sequence of HBD and non-HBD segments, the Viterbi algorithm was applied, which is a dynamic programming algorithm proposed by Andrew Viterbi in 1967 and discussed in detail by L.R. Rabiner [16]. Transition probabilities were modeled taking into account the fact that the length of HBD segments is exponentially distributed. HBD segments were divided into *k* classes (*k* = 10). Each HBD class has its own expected length and frequency, which allows it to correspond to more realistic situations where the ancestors that promote autozygosity date back to different generations in the past. The probability of a segment termination between two markers separated by d Morgans is $e^{-R_{k}d}$ ; where $R_{k}$ is the exponential distribution rate for each class, so the expected length of HBD segments is then 1/$-R_{k}$ Morgans (higher values correspond to shorter segments) [14,17].

The coefficients of Rk were set from 2 to 512 (2,4,8,16,32,64,128,256,512) to achieve more classes for long HBD segments. For example, the class with a coefficient of Rk = 2 roughly corresponds to the ancestors of one generation ago (parents), the class with a coefficient of Rk = 4 approximately corresponds to the ancestors of two generations ago (grandparents) and the class with a coefficient of Rk = 512 corresponds to the ancestors of approximately 206 generations ago. The assessments of each class in autozygosity are difficult to interpret as inbreeding coefficients, since they have variability in an individual, and in this case, to obtain an inbreeding coefficient, we summarized the autozygosity for all HBD classes (it can be calculated as 1 minus the proportion of non-HBD). 

A search of QTL, genes and deleterious variations (SIFT) was performed in the Ensembl genome browser (Sscrofa 11.1) (https://www.ensembl.org/index.html accessed on 12 July 2021).

## 3. Results

**Figure 1:**
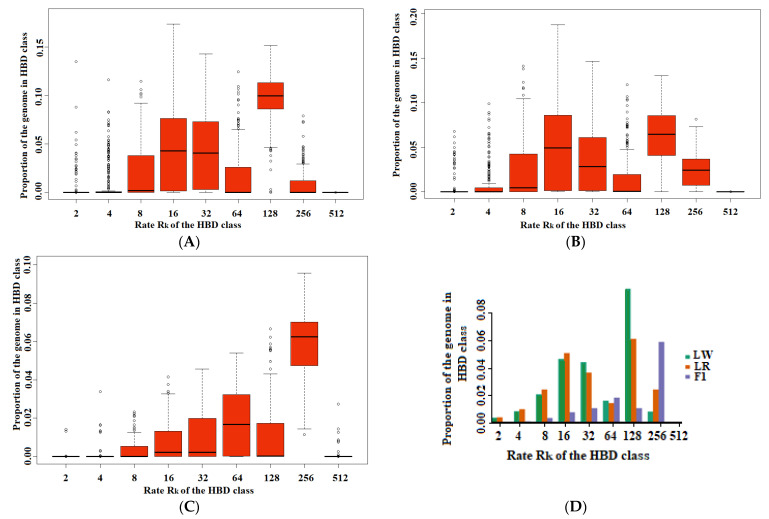
Autozygosity of pigs depending on the rate of Rk of the HBD class ((**A**)—LW; (**B**)—LR; (**C**)—F1; (**D**)—LW, LR and F1).

The realized autozygosity in purebred animals was 0.24 in LW and 0.22 in LR (non-HBD class is less than 0.80). In F1, the non-HBD class was approximately 0.89, and the autozygosity was 0.11% of the genome, respectively. The class with the coefficient Rk_128 made the greatest contribution to the realized autozygosity in purebred livestock (Figure 1). In this case, the contribution of the class Rk_128 for LW was approximately 0.097, and for LR, approximately 0.061. In F1, the class with the coefficient Rk_256 (approximately 0.059) made the greatest contribution to autozygosity.

**Figure 2:**
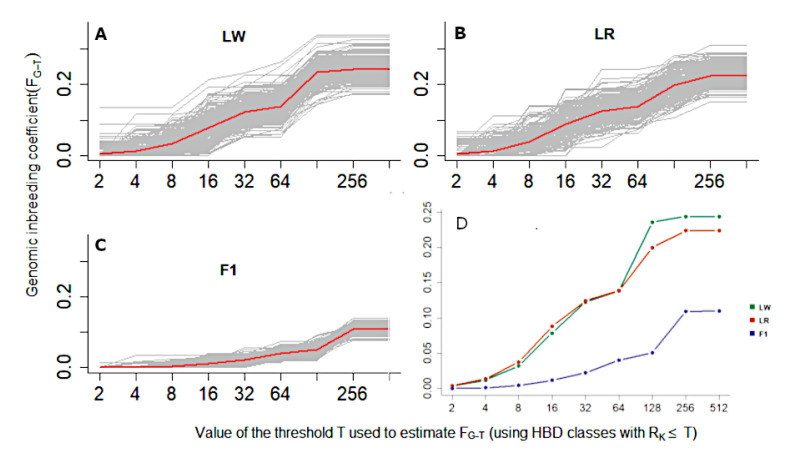
Variations in individual levels of autozygosity accumulated across all HBD classes ((**A**)—LW; (**B**)—LR; (**C**)—F1; (**D**)—LW, LR and F1).

Variations in individual levels of autozygosity accumulated across all HBD classes (summation of total autozygosity associated with HBD classes) are shown in Figure 2.

**Figure 3:**
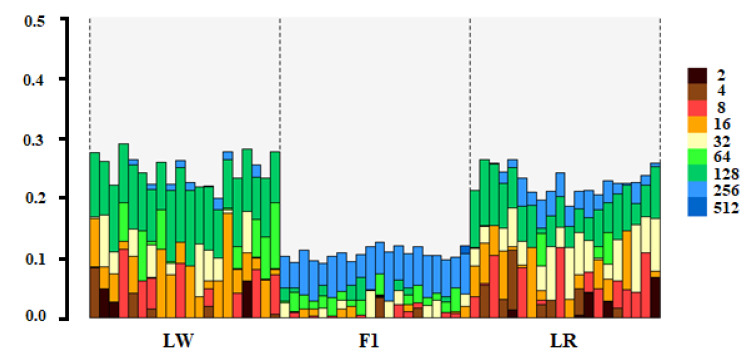
Genome division in different classes of homozygous origin (HBD) in Landrace, Large White and crossbred F1.

It is obvious that for different individuals with the same general inbreeding, the contribution of partial inbreeding of different ages can be very different. This can be seen in Figure 3, which shows the HBD grades in randomly selected pigs: LW (*n* = 20), LR (*n* = 20) and F1 (*n* = 20). The height of each bar represents the fraction of the genome associated with the HBD class of the corresponding color.

**Figure 4:**
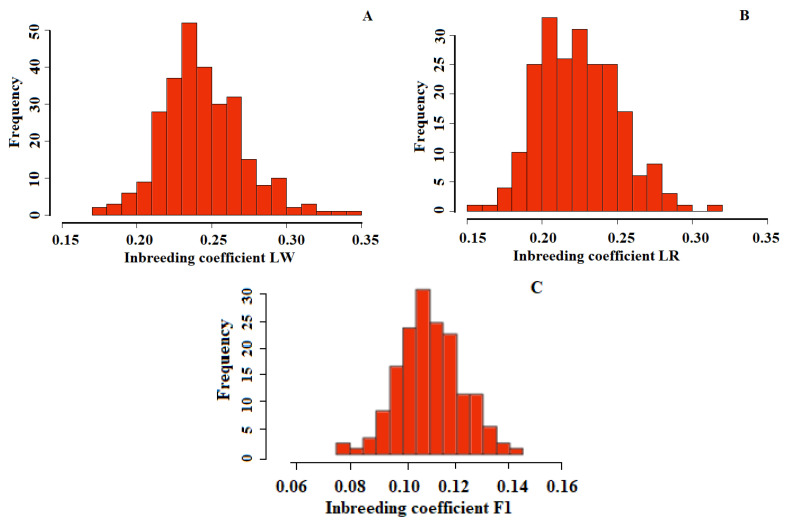
Summary statistics of the inbreeding coefficient (calculated as the sum of the contributions of all HBD classes) ((**A**)—LW, (**B**)—LR, (**C**)—F1).

In this regard, the estimates of each class in autozygosity are difficult to interpret as inbreeding coefficients, since they have individual variability, and in this case, to obtain the inbreeding coefficient, we summarized the autozygosity for all HBD classes. The implemented autozygosity for all HBD classes (inbreeding coefficient) in crossbred animals decreased twice as much as purebred animals (Figure 4, Table 1).

Using the Viterbi algorithm, we identified the HBD segments. In LW, 50,420 HBD segments were identified, with an average of 180 per animal; in LR, 33,586 HBD segments were identified, with an average of 154 per animal; in F1 pigs, 21,068 HBD segments were identified, with an average of 132 per animal. The largest length of HBD segments was determined in LW pigs (203.93 Mb, 3784 SNP number, SCC 13). The average length of HBD segments in purebred pigs was approximately 3.20–3.37 Mb (78–83 SNP number), and in crossbred pigs, 1.70 Mb (39.58 SNP number).

In general, all pigs have a large number of short segments belonging to distant ancestors. Thus, in LW and LR, more than 50% of the HBD segments (35,254 number HBD for LW; 17,145 number HBD for LR) belonged to the Rk_128 class, and in F1, approximately 70% of the HBD segments (14,889) belonged to the Rk_256 class (Table 2).

**Figure 5:**
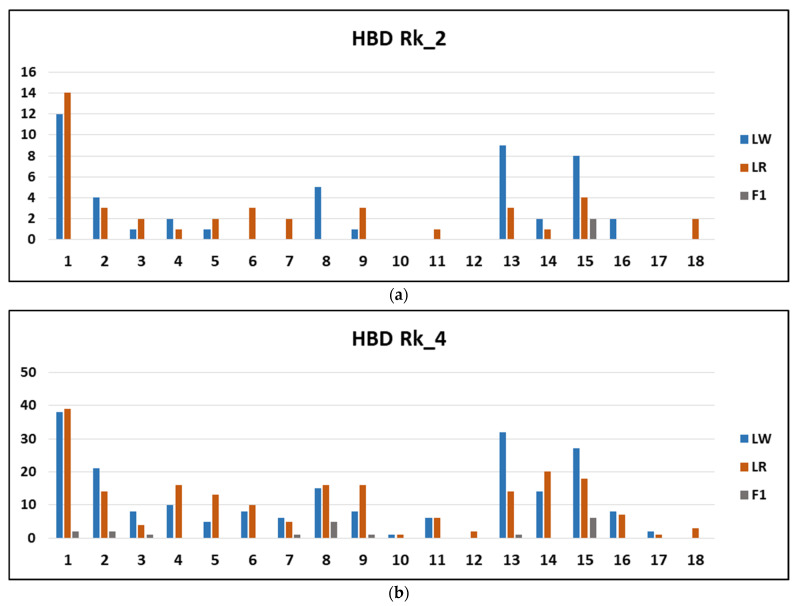
Distribution by chromosome of the longest HBD segments belonging to classes Rk_2 and Rk_4.

The distribution by chromosome of the longest HBD segments belonging to classes Rk_2 and Rk_4 is shown in Figure 5. In LW, the HBD segments Rk_2 and Rk_4 are most represented on SSC1, SSC13 and SSC15; in LR, they are most represented on SSC1. In F1 pigs, there are only 21 segments belonging to the HBD Rk_2 and Rk_4 classes, of which 8 segments are localized in SC15.

It is assumed that long sections of consecutive homozygous genotypes contain variants associated with important economic traits, but they are also enriched with deleterious variations and, accordingly, contribute to the maintenance of both useful and deleterious variations in the population. For the analysis, we selected several regions of the genome with the longest HBD segments: SSC1 (183.7 Mb), which is enriched in HBD segments in both LW and LR; SSC13 (203.93 Mb), with the longest segment in the LW; and SSC15 (122.64 Mb), which is enriched with HBD segments in LW, LR and F1. These areas overlap with 441 QTLs (SSC1—238 QTLs; SSC13—101 QTLs; and SSC15—102 QTLs), including 174 QTLs for meat and carcass traits (84 QTLs—fatness), 127 QTLs for reproduction traits (100 QTLs—litter traits), 101 QTLs for production traits (69 QTLs—growth and 30 QTLs—feed intake), 21 QTLs for exterior traits (9 QTLs—conformation) and 18 QTLs for health traits (13 QTLs—blood parameters) (Supplementary Table S1). In addition to QTLs, mutations were identified in these areas, presented in the catalog/compendium of inherited disorders OMIA: OMIA 001718-9823: dwarfism and Schmid metaphyseal chondrodysplasia in Sus scrofa (DIAS0001377, SSC1); OMIA 002210-9823: hypothyroidism, congenital and DUOX2-related disorders in Sus scrofa (MARC0108203, SSC1); OMIA 001401-9823: Waardenburg syndrome and type 2A in Sus scrofa (ASGA0057575, SSC13).

**Figure 6:**
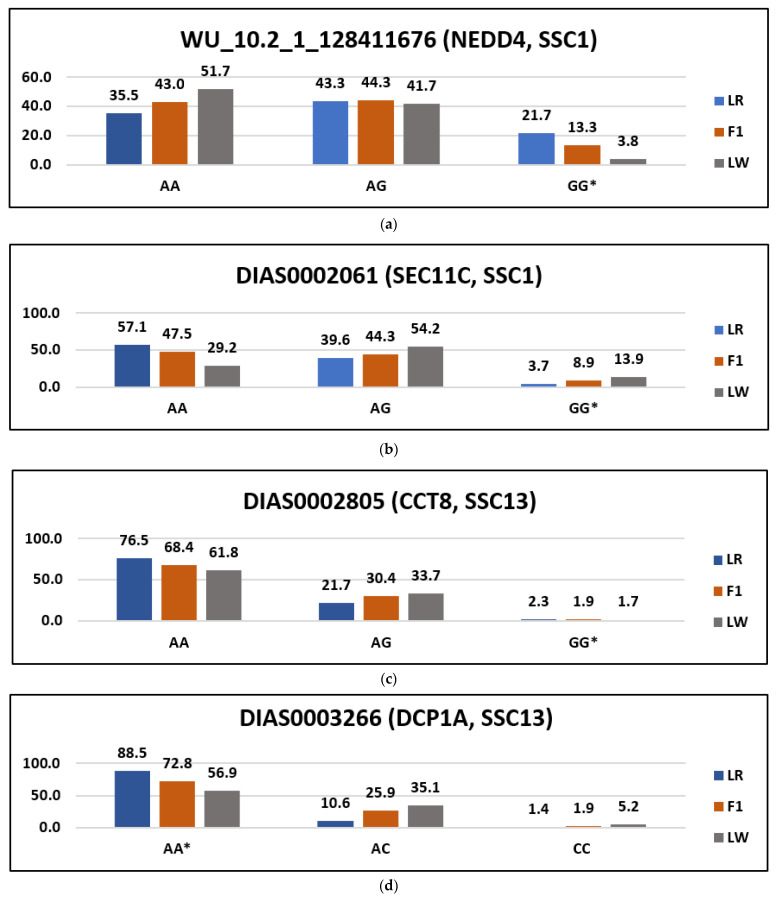

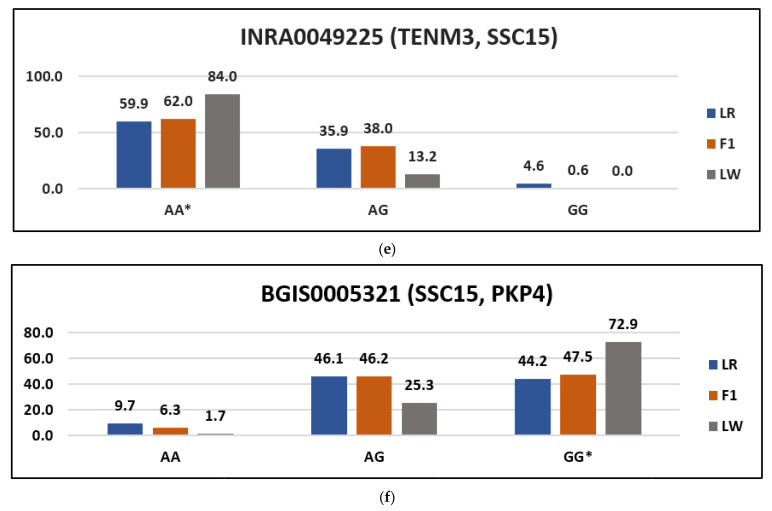
Frequency of the possibly deleterious variants (*—homozygous genotype for a possibly deleterious variant).

It is traditionally believed that point mutations in gene exons exhibit their effects by altering amino acids in encoded proteins [18]. In the HBD segments under study, 3898 SNPs localized in 1252 genes were identified, 30 of which were missense variants (Table 3). When assessing the possibility of deleterious variation in these variants, we identified six SNPs, out of which two were tolerated low confidence, WU_10.2_1_128411676 (NEDD4, SSC1) and BGIS0005321 (PKP4, SSC15); one was SNP deleterious low confidence, DIAS0002061 (SEC11C, SSC1); and three were SNP deleterious, DIAS0003266 (DCP1A, SSC13), DIAS0002805 (CCT8, SSC13) and INRA0049225 (TENM3, SSC15). The frequencies of these variants are shown in Figure 6.

## 4. Discussion

Organized pig breeding in Europe dates back to the 18th century [19,20]. The nucleus in forming the Large White breed was the local marching pig, which was improved by the Romanov, Eastern and Chinese pigs (the first import of Chinese pigs dates back to 1770–1780) [21]. Initially, pigs were called Yorkshires, and in 1885, they were named the Large White breed. Subsequently, the Large White participated in forming and improving most modern European breeds, including Landrace. In the first half of the 19th century, the import of pigs began in Denmark from Germany, Portugal, China, Spain and England, which contributed to the improvement of local Celtic-type pigs [22]. By targeted selection at the end of the 19th century, the Landrace breed was created in Denmark. In recent decades, the commercial pig breeding industry has triggered significant changes in selection programs with a focus on the cost effectiveness of production [23,24,25,26,27,28].

Purebred animals must have homozygous regions conditioning some level of inbreeding. Our studies showed that in LR and LW pigs, total autozygosity (inbreeding coefficient) was approximately 0.23 (0.15–0.34), and in F1 pigs, it significantly decreased to 0.11 (0.08–0.14). In this study, the Rk_128 of the HBD class made the greatest contribution to the autozygosity of LW and LR, which is approximately 64 generations ago, and in F1, accordingly, the Rk_256 of the HBD class made the greatest contribution to autozygosity. This is possibly connected with the development of some breeding programs and the targeted selection of pigs with definite productivity. It is difficult to interpret the exact time intervals, but it can be noted that the period of 1900–1945 in European countries is characterized by an organized and nationally controlled breeding system for purebred pigs and the creation of breeding centers [22].

We can also note the contribution to autozygosity in LR and LW pigs of the Rk_16 and Rk_32 classes (approximately 8–16 generations ago), which is possibly associated with the development of the commercial pig breeding industry. In recent decades, the pig industry has experienced significant consolidation, resulting in mergers and acquisitions of breeding companies. Breeding lines have united, thereby forming new “breeding nuclei” of international leading swine genetics companies [25].

In any case, based on the data obtained in our work, we can only build hypotheses, since more animals from various breeding cents are needed. However, using the approach based on the HBD multiple class model proposed by Druet and Gaultier [14], we can trace the history of the population to determine the areas subjected to selection pressure in a particular period. In this aspect, long segments of HBD are of great interest for animal husbandry, since they reflect more recent events in the population. Additionally, it is believed that in the long sections of homozygosity, deleterious variants will become more common, although it is emphasized that there are harmless allelic variants with a high frequency [29,30,31].

In this work, we focused only on the longest HBD segments identified in LR and LW. In our study, in the studied pig populations, long segments of HBD were identified on chromosomes SSC1, SSC13 and SSC15. The results showed that QTLs associated with the most significant breeding traits are localized in these areas, such as average daily gain, daily feed intake, average backfat thickness, intramuscular fat content, total number born and teat number. However, SNPs have also been identified in these areas that overlap the areas presented in the OMIA catalog/compendium of inherited disorders. OMIA 001718-9823 is associated with dwarfism and Schmid metaphyseal chondrodysplasia in Sus scrofa, and is an autosomal dominant mutation that causes dwarfism with metaphyseal chondrodysplasia in pigs, and metaphisms can be seen in the thoracic vertebrae, shoulder blades, metatarsal and metacarpal bones [32]. OMIA 002210-9823 is associated with hypothyroidism and congenital and DUOX2-related disorders in Sus scrofa, and may play a role in severe thyroid hormone deficiency in pigs [33]. OMIA 001401-9823 is associated with Waardenburg syndrome and type 2A in Sus scrofa, and is an autosomal recessive mutation associated with hearing loss in pigs [34].

Using the SIFT server, we analyzed whether the SNPs, localized in long segments of HBD, have a harmful or damaging effect on the function of the SNPs protein. This resulted in identification of six SNPs localized in the NEDD4, SEC11C, DCP1A, CCT8, PKP4 and TENM3 genes, which may be deleterious variants. The NEDD4 gene is involved in regulating the water–electrolyte balance by controlling the number of sodium channels in epithelial cells. Studies have also shown that NEDD4 acts as an E3 ligase and regulates the embryonic development and growth of animals (proliferation, autophagy and differentiation of multiple malignancies) [35]. The TENM3 gene encodes highly conservative transmembrane glycoproteins of type II that are widely expressed in the nervous system and play a key role in regulating the development of the nervous system [36]. Feldman et al. [37] proposed that mutations in the TENM3 gene in humans and mice lead to a slowdown of chondrogenes. Singh et al. [38] also associated variations of this gene with eye abnormalities (microphthalmia and anophthalmia) and mental retardation in humans. The PKP4 gene is involved in the regulation of cadherin function. Schröder et al. [39] in their study proved that the PKP4 gene is an integral part of the contractile apparatus in human skeletal muscles. The SEC11 gene encodes a subunit of the signal peptidase complex. The DCP1A gene encodes a protein that is the main component in the processes of mRNA decapitation and degradation [40], and it also participates as a transcriptional co-activator in the SMAD4-TGF-β pathway [41]. The CCT8 gene encodes a molecular chaperone, the main role of which is to ensure the correct stacking of proteins. Noormohammadi et al. [42] presented the role of CCT8 as a powerful candidate for maintaining proteostasis during organism aging.

The role played by the variants of the genes NEDD4, SEC11C, DCP1A, CCT8, PKP4 and TENM3 in pig phenotypes has not been published in other studies to date. We can note that in our sample, a high frequency of potential deleterious variants was noted for LR on the DCP1A gene, and for LW on TENM3 and PKP4. In all cases, frequencies of genotypes at F1 occupied intermediate values between LR and LW, which indicates the possibility to consider the potential decrease in the negative influence of harmful variants concerning thoroughbred pigs.

## 5. Conclusions

This paper presents an analysis of segments HBD and non-HBD in purebred and crossbred livestock of pigs. Here, we should note that when we use the technology of genotyping, many SNPs are also localized in introns and intergenic regions. This fact imposes restrictions on the functional analysis of HBD segment sequences. However, the findings presented in our work show promise for the genome scanning of HBD as a strategy for studying population history, and identifying genomic regions and genes associated with important economic traits and deleterious variants.

## Figures and Tables

**Table 1:** Summary statistics of the inbreeding coefficient.

	LW	LR	F1
Min	0.17	0.15	0.08
1st Qu.	0.27	0.20	0.10
Median	0.24	0.22	0.11
Mean	0.24	0.22	0.11
3rd Qu.	0.26	0.24	0.12
Max.	0.34	0.31	0.14

**Table 2:** Number and length (Mb) of the HBD segments.

Rk	LW		LR		F1	
	Number	Length	Number	Length	Number	Length
2	47	76.286 ± 5.120	41	73.447 ± 5.486	2	63.836 ± 1.797
4	209	41.175 ± 1.463	205	38.593 ± 1.491	19	32.509 ± 3.605
8	1042	20.225 ± 0.362	864	20.204 ± 0.414	128	19.471 ± 0.715
16	4589	9.576 ± 0.089	3910	9.088 ± 0.096	446	10.497 ± 0.303
32	5820	5.356 ± 0.032	3873	4.725 ± 0.040	1362	4.837 ± 0.072
64	2349	3.068 ± 0.026	2176	2.697 ± 0.027	2679	3.030 ± 0.024
128	35,254	1.283 ± 0.004	17,145	1.292 ± 0.006	1543	1.537 ± 0.019
256	1114	0.554 ± 0.009	5372	0.572 ± 0.004	14,889	0.732 ± 0.003

**Table 3:** Missense variants in the longest HBD segments.

Uploaded_Variation	Position	Allele	Symbol	Amino_Acids	Codons	Variation	SIFT
WU_10.2_1_128411676	1:115993957	G	*NEDD4*	I/V	Ata/Gta	rs331958194	tolerated_low_confidence (1)
DIAS0002234	1:29042621	G	*HBS1L*	I/V	Att/Gtt	rs80787670	Tolerated (1)
MARC0008887	1:95536583	C	*EPG5*	I/V	Atc/Gtc	rs81253573	Tolerated (1)
MARC0005035	1:108340862	G	*HERC1*	N/S	aAc/aGc	rs80998094	Tolerated (1)
WU_10.2_1_202674735	1:182404193	T	*STYX*	S/L	tCg/tTg	rs321526744	Tolerated (1)
DRGA0001262	1:82324692	A	*-*	E/K	Gaa/Aaa	rs80984416	Tolerated (0.71)
ALGA0002996	1:44498050	G	*ROS1*	D/G	gAt/gGt	rs80883735	Tolerated (0.65)
DIAS0002722	1:25558722	A	*REPS1*	G/D	gGt/gAt	rs333867086	Tolerated (0.64)
DIAS0002980	1:26316436	C	*PERP*	T/P	Act/Cct	rs55618815	Tolerated (0.39)
ASGA0004152	1:106877209	G	*FECH*	V/A	gTg/gCg	rs81216562	Tolerated (0.22)
DIAS0003245	1:179469474	A	*LRR1*	E/K	Gaa/Aaa	rs332693293	Tolerated (0.13)
DIAS0002061	1:161757995	G	*SEC11C*	C/R	Tgt/Cgt	rs80807772	deleterious_low_confidence (0)
M1GA0025601	13:34117528	A	*-*	V/I	Gta/Ata	rs81478482	Tolerated (1)
DIAS0004147	13:106612102	A	*SERPINI1*	L/I	Ctt/Att	rs322745111	Tolerated (1)
WU_10.2_13_42333381	13:38487829	C	*CCDC66*	I/T	aTa/aCa	rs335407407	Tolerated (0.52)
DIAS0001169	13:13969030	G	*SLC4A7*	T/P	Act/Cct	rs339676777	Tolerated (0.5)
INRA0040732	13:89489538	G	*-*	I/T	aTc/aCc	rs345909418	Tolerated (0.5)
M1GA0025255	13:29119930	T	*FYCO1*	D/N	Gac/Aac	rs81478691	Tolerated (0.47)
DIAS0003138	13:22199433	A	*GOLGA4*	E/K	Gaa/Aaa	rs328179266	Tolerated (0.31)
DIAS0003446	13:24649204	A	*ENTPD3*	A/T	Gca/Aca	rs81216415	Tolerated (0.25)
DBMA0000259	13:122067534	T	*EIF2B5*	T/M	aCg/aTg	rs45435374	Tolerated (0.22)
DIAS0002680	13:73754891	A	*ACAD11*	A/V	gCa/gTa	rs326329989	Tolerated (0.14)
DIAS0003266	13:35402355	T	*DCP1A*	A/D	gCc/gAc	rs81211881	Deleterious (0.04)
DIAS0002805	13:192415347	G	*CCT8*	S/P	Tct/Cct	rs81214915	Deleterious (0)
BGIS0005321	15:65680055	G	*PKP4*	I/V	Ata/Gta	rs80939022	tolerated_low_confidence (1)
DIAS0001114	15:72586615	T	*SCN1A*	R/K	aGg/aAg	rs340033396	Tolerated (1)
DIAS0000678	15:121561503	A	*OBSL1*	A/S	Gct/Tct	rs332398561	Tolerated (0.79)
MARC0063762	15:106573135	A	*CYP20A1*	R/Q	cGg/cAg	rs81252138	Tolerated (0.64)
MARC0035976	15:44768162	T	*WWC2*	P/S	Ccc/Tcc	rs81230064	Tolerated (0.28)
INRA0049225	15:44344760	A	*TENM3*	V/M	Gtg/Atg	rs345636277	Deleterious (0)

## Data Availability

The raw data supporting the conclusions of this article will be made available by the authors upon reasonable request.

## Supplementary Materials

The following are available online at https://www.mdpi.com/article/10.3390/life11080861/s1, Supplementary Table S1: QTLs in the longest HBD segments.
