# Supplementary material for: Analysis of Homozygous-by-Descent (HBD) Segments for Purebred and Crossbred Pigs in Russia
Source: Life (Basel). 2021 Aug 22;11(8):861. doi: 10.3390/life11080861 (PMC8400874; doi:10.3390/life11080861)
Supplement: Supplementary file 1 [file life-11-00861-s001.zip › life-1289085-supplementary.pdf]

Supp. Table S1. QTLs in the longest HBD segments.

ID QTL	SSC	Trait	Class	Group
22910	1	Total_number_born	Litter traits	Reproduction traits
18485	1	Total_number_born	Litter traits	Reproduction traits
18219	1	Total_number_born	Litter traits	Reproduction traits
18218	1	Total_number_born	Litter traits	Reproduction traits
18070	1	Total_number_born_alive	Litter traits	Reproduction traits
18115	1	Number_of_stillborn	Litter traits	Reproduction traits
18291	1	mummified_pigs	Litter traits	Reproduction traits
18290	1	mummified_pigs	Litter traits	Reproduction traits
18272	1	mummified_pigs	Litter traits	Reproduction traits
18270	1	mummified_pigs	Litter traits	Reproduction traits
18239	1	mummified_pigs	Litter traits	Reproduction traits
106223	1	Litter_size	Litter traits	Reproduction traits
15972	1	Lifetime_number_born_alive	Litter traits	Reproduction traits
31822	1	Corpus_luteum_number	Litter traits	Reproduction traits
22911	1	Total_number_born	Litter traits	Reproduction traits
22921	1	Total_number_born_alive	Litter traits	Reproduction traits
22935	1	Litter_weight__total	Litter traits	Reproduction traits
18271	1	mummified_pigs	Litter traits	Reproduction traits
178884	1	Number_of_mummified_pigs	Litter traits	Reproduction traits
178883	1	Number_of_mummified_pigs	Litter traits	Reproduction traits
178885	1	Number_of_mummified_pigs	Litter traits	Reproduction traits
18128	1	Number_of_stillborn	Litter traits	Reproduction traits
18490	1	Litter_weight__piglets_born_alive	Litter traits	Reproduction traits
18492	1	Litter_weight__piglets_born_alive	Litter traits	Reproduction traits
15973	1	Lifetime_total_number_born	Litter traits	Reproduction traits
15974	1	Lifetime_total_number_born	Litter traits	Reproduction traits
15975	1	Lifetime_number_born_alive	Litter traits	Reproduction traits

ID QTL	SSC	Trait	Class	Group
22922	1	Total_number_born_alive	Litter traits	Reproduction traits
57514	1	Litter_birth_interval	Reprodictive traits	Reproduction traits
96089	1	Age_at_puberty	Reprodictive traits	Reproduction traits
18167	1	Gestation_length	Reprodictive traits	Reproduction traits
126635	1	Teat_number	Reproductive organ	Reproduction traits
126623	1	Teat_number	Reproductive organ	Reproduction traits
126650	1	Teat_number__difference_between_sides	Reproductive organ	Reproduction traits
126589	1	Teat_number__difference_between_sides	Reproductive organ	Reproduction traits
126633	1	Left_teat_number	Reproductive organ	Reproduction traits
211855	1	Teat_number	Reproductive organ	Reproduction traits
211788	1	Teat_number	Reproductive organ	Reproduction traits
211787	1	Teat_number	Reproductive organ	Reproduction traits
211786	1	Teat_number	Reproductive organ	Reproduction traits
126713	1	Teat_number	Reproductive organ	Reproduction traits
29553	1	Feed_conversion_ratio	Feed conversion	Production traits
29552	1	Feed_conversion_ratio	Feed conversion	Production traits
66267	1	Daily_feed_intake	Feed intake	Production traits
29543	1	Daily_feed_intake	Feed intake	Production traits
22198	1	Daily_feed_intake	Feed intake	Production traits
135641	1	Daily_feed_intake	Feed intake	Production traits
135639	1	Daily_feed_intake	Feed intake	Production traits
22427	1	Average_feeding_rate	Feed intake	Production traits
22386	1	Daily_feed_intake	Feed intake	Production traits
22385	1	Daily_feed_intake	Feed intake	Production traits
22389	1	Daily_feed_intake	Feed intake	Production traits
22388	1	Daily_feed_intake	Feed intake	Production traits
22387	1	Daily_feed_intake	Feed intake	Production traits
22384	1	Daily_feed_intake	Feed intake	Production traits
22370	1	Daily_feed_intake	Feed intake	Production traits

ID QTL	SSC	Trait	Class	Group
135650	1	Time_in_feeder_per_day	Feed intake	Production traits
29544	1	Daily_feed_intake	Feed intake	Production traits
135640	1	Daily_feed_intake	Feed intake	Production traits
135651	1	Time_in_feeder_per_day	Feed intake	Production traits
135649	1	Time_in_feeder_per_day	Feed intake	Production traits
22196	1	Daily_feed_intake	Feed intake	Production traits
22205	1	Daily_feed_intake	Feed intake	Production traits
135638	1	Daily_feed_intake	Feed intake	Production traits
135648	1	Time_in_feeder_per_day	Feed intake	Production traits
22209	1	Daily_feed_intake	Feed intake	Production traits
29545	1	Daily_feed_intake	Feed intake	Production traits
22210	1	Daily_feed_intake	Feed intake	Production traits
22211	1	Daily_feed_intake	Feed intake	Production traits
139161	1	Da+45:84ys_to_110_kg	Growth	Production traits
66275	1	Body_weight	Growth	Production traits
65010	1	Body_weight	Growth	Production traits
65008	1	Body_weight	Growth	Production traits
22971	1	Body_weight_birth	Growth	Production traits
22970	1	Body_weight_birth	Growth	Production traits
172209	1	Body_height	Growth	Production traits
22269	1	Average_daily_gain	Growth	Production traits
22232	1	Average_daily_gain	Growth	Production traits
22222	1	Average_daily_gain	Growth	Production traits
22268	1	Average_daily_gain	Growth	Production traits
22221	1	Average_daily_gain	Growth	Production traits
28800	1	Average_daily_gain	Growth	Production traits
28801	1	Average_daily_gain	Growth	Production traits
28802	1	Average_daily_gain	Growth	Production traits
28803	1	Average_daily_gain	Growth	Production traits

ID QTL	SSC	Trait	Class	Group
28804	1	Average_daily_gain	Growth	Production traits
28799	1	Average_daily_gain	Growth	Production traits
28798	1	Average_daily_gain	Growth	Production traits
28797	1	Average_daily_gain	Growth	Production traits
28796	1	Average_daily_gain	Growth	Production traits
22267	1	Average_daily_gain	Growth	Production traits
22220	1	Average_daily_gain	Growth	Production traits
22263	1	Average_daily_gain	Growth	Production traits
22264	1	Average_daily_gain	Growth	Production traits
22216	1	Average_daily_gain	Growth	Production traits
22217	1	Average_daily_gain	Growth	Production traits
28179	1	Average_daily_gain_on_test	Growth	Production traits
22265	1	Average_daily_gain	Growth	Production traits
22219	1	Average_daily_gain	Growth	Production traits
22218	1	Average_daily_gain	Growth	Production traits
22266	1	Average_daily_gain	Growth	Production traits
28795	1	Average_daily_gain	Growth	Production traits
28178	1	Average_daily_gain_entire_life	Growth	Production traits
22233	1	Average_daily_gain	Growth	Production traits
29535	1	Average_daily_gain_on_test	Growth	Production traits
29533	1	Average_daily_gain_on_test	Growth	Production traits
211957	1	Body_weight_birth	Growth	Production traits
22235	1	Average_daily_gain	Growth	Production traits
22234	1	Average_daily_gain	Growth	Production traits
65130	1	Body_weight	Growth	Production traits
22261	1	Average_daily_gain	Growth	Production traits
211924	1	Days_to_90_kg	Growth	Production traits
22260	1	Average_daily_gain	Growth	Production traits
211901	1	Days_to_90_kg	Growth	Production traits

ID QTL	SSC	Trait	Class	Group
22258	1	Average_daily_gain	Growth	Production traits
22259	1	Average_daily_gain	Growth	Production traits
139164	1	Days_to_110_kg	Growth	Production traits
139163	1	Days_to_110_kg	Growth	Production traits
22262	1	Average_daily_gain	Growth	Production traits
66268	1	Body_weight	Growth	Production traits
29534	1	Average_daily_gain_on_test	Growth	Production traits
22226	1	Average_daily_gain	Growth	Production traits
22227	1	Average_daily_gain	Growth	Production traits
22228	1	Average_daily_gain	Growth	Production traits
22229	1	Average_daily_gain	Growth	Production traits
139162	1	Days_to_110_kg	Growth	Production traits
22224	1	Average_daily_gain	Growth	Production traits
22225	1	Average_daily_gain	Growth	Production traits
29579	1	Loin_muscle_depth	Anatomy	Meat and carcass trait
22332	1	Loin_muscle_area	Anatomy	Meat and carcass trait
22329	1	Loin_muscle_area	Anatomy	Meat and carcass trait
65013	1	Lean_meat_weight	Anatomy	Meat and carcass trait
95695	1	Ham_weight	Anatomy	Meat and carcass trait
95694	1	Ham_weight	Anatomy	Meat and carcass trait
95693	1	Ham_weight	Anatomy	Meat and carcass trait
95692	1	Ham_weight	Anatomy	Meat and carcass trait
28022	1	Belly_weight	Anatomy	Meat and carcass trait
22356	1	Loin_muscle_area	Anatomy	Meat and carcass trait
22340	1	Loin_muscle_area	Anatomy	Meat and carcass trait
22355	1	Loin_muscle_area	Anatomy	Meat and carcass trait
22357	1	Loin_muscle_area	Anatomy	Meat and carcass trait
22341	1	Loin_muscle_area	Anatomy	Meat and carcass trait
22358	1	Loin_muscle_area	Anatomy	Meat and carcass trait

ID QTL	SSC	Trait	Class	Group
22342	1	Loin_muscle_area	Anatomy	Meat and carcass trait
22359	1	Loin_muscle_area	Anatomy	Meat and carcass trait
22337	1	Loin_muscle_area	Anatomy	Meat and carcass trait
22339	1	Loin_muscle_area	Anatomy	Meat and carcass trait
22338	1	Loin_muscle_area	Anatomy	Meat and carcass trait
22352	1	Loin_muscle_area	Anatomy	Meat and carcass trait
22353	1	Loin_muscle_area	Anatomy	Meat and carcass trait
22336	1	Loin_muscle_area	Anatomy	Meat and carcass trait
22351	1	Loin_muscle_area	Anatomy	Meat and carcass trait
22354	1	Loin_muscle_area	Anatomy	Meat and carcass trait
211925	1	Loin_muscle_area	Anatomy	Meat and carcass trait
17771	1	Loin_muscle_area	Anatomy	Meat and carcass trait
22343	1	Loin_muscle_area	Anatomy	Meat and carcass trait
22344	1	Loin_muscle_area	Anatomy	Meat and carcass trait
22333	1	Loin_muscle_area	Anatomy	Meat and carcass trait
22345	1	Loin_muscle_area	Anatomy	Meat and carcass trait
22334	1	Loin_muscle_area	Anatomy	Meat and carcass trait
22335	1	Loin_muscle_area	Anatomy	Meat and carcass trait
193143	1	Loin_weight	Anatomy	Meat and carcass trait
193144	1	Ham_weight	Anatomy	Meat and carcass trait
18654	1	androstenone__laboratory	Chemistry	Meat and carcass trait
161322	1	Conductivity_45_minutes_post-mortem	Conductivity impedance	Meat and carcass trait
17772	1	Conductivity_45_minutes_post-mortem	Conductivity impedance	Meat and carcass trait
65015	1	Lean_meat_percentage	Fatness	Meat and carcass trait
139172	1	Lean_meat_percentage	Fatness	Meat and carcass trait
29591	1	Intramuscular_fat_content	Fatness	Meat and carcass trait
193140	1	Intramuscular_fat_content	Fatness	Meat and carcass trait
125483	1	Intramuscular_fat_content	Fatness	Meat and carcass trait
65012	1	Fat_weight_total	Fatness	Meat and carcass trait

ID QTL	SSC	Trait	Class	Group
65009	1	Fat_weight_total	Fatness	Meat and carcass trait
65007	1	Fat_weight_total	Fatness	Meat and carcass trait
65014	1	Fat_percentage_in_carcass	Fatness	Meat and carcass trait
65011	1	Fat_percentage_in_carcass	Fatness	Meat and carcass trait
121914	1	Backfat_between_3rd_and_4th_last_ribs	Fatness	Meat and carcass trait
121909	1	Backfat_between_3rd_and_4th_last_ribs	Fatness	Meat and carcass trait
29564	1	Average_backfat_thickness	Fatness	Meat and carcass trait
29531	1	Average_backfat_thickness	Fatness	Meat and carcass trait
211916	1	Average_backfat_thickness	Fatness	Meat and carcass trait
193553	1	Intramuscular_fat_content	Fatness	Meat and carcass trait
193554	1	Intramuscular_fat_content	Fatness	Meat and carcass trait
28180	1	Fat_area	Fatness	Meat and carcass trait
28181	1	Backfat_at_last_rib	Fatness	Meat and carcass trait
139173	1	Lean_meat_percentage	Fatness	Meat and carcass trait
211930	1	Lean_meat_percentage	Fatness	Meat and carcass trait
17773	1	Average_backfat_thickness	Fatness	Meat and carcass trait
65016	1	Fat_percentage_in_carcass	Fatness	Meat and carcass trait
65018	1	Fat_weight_total	Fatness	Meat and carcass trait
29565	1	Average_backfat_thickness	Fatness	Meat and carcass trait
32099	1	Palmitic_acid_content	Fatty acid content	Meat and carcass trait
32098	1	Palmitic_acid_content	Fatty acid content	Meat and carcass trait
32063	1	Palmitic_acid_content	Fatty acid content	Meat and carcass trait
32062	1	Palmitic_acid_content	Fatty acid content	Meat and carcass trait
106264	1	Monounsaturated_fatty_acid_content	Fatty acid content	Meat and carcass trait
32103	1	Linoleic_acid_content	Fatty acid content	Meat and carcass trait
32102	1	Linoleic_acid_content	Fatty acid content	Meat and carcass trait
32101	1	Linoleic_acid_content	Fatty acid content	Meat and carcass trait
32100	1	Linoleic_acid_content	Fatty acid content	Meat and carcass trait
32065	1	Eicosatrienoic_acid_content	Fatty acid content	Meat and carcass trait

ID QTL	SSC	Trait	Class	Group
22470	1	Stearic_acid_content	Fatty acid content	Meat and carcass trait
22469	1	cis-11-Eicosenoic_acid_content	Fatty acid content	Meat and carcass trait
193486	1	Stearic_acid_content	Fatty acid content	Meat and carcass trait
193482	1	Palmitoleic_acid_to_palmitic_acid_ratio	Fatty acid content	Meat and carcass trait
193481	1	Palmitoleic_acid_to_palmitic_acid_ratio	Fatty acid content	Meat and carcass trait
193480	1	Palmitoleic_acid_to_palmitic_acid_ratio	Fatty acid content	Meat and carcass trait
193479	1	Palmitoleic_acid_to_palmitic_acid_ratio	Fatty acid content	Meat and carcass trait
193475	1	Palmitoleic_acid_to_palmitic_acid_ratio	Fatty acid content	Meat and carcass trait
193483	1	Stearic_acid_content	Fatty acid content	Meat and carcass trait
193478	1	Palmitoleic_acid_to_palmitic_acid_ratio	Fatty acid content	Meat and carcass trait
121898	1	Meat_color_b*	Meat color	Meat and carcass trait
95258	1	Meat_color_a*	Meat color	Meat and carcass trait
193141	1	Meat_color_L*	Meat color	Meat and carcass trait
193142	1	Meat_color_b*	Meat color	Meat and carcass trait
28046	1	pH_24_hr_post_mortem_ham	pH	Meat and carcass trait
17770	1	pH_24_hr_post_mortem_ham	pH	Meat and carcass trait
28105	1	pH_24_hr_post_mortem_ham	pH	Meat and carcass trait
22433	1	Shear_force	Texture	Meat and carcass trait
28107	1	Water_holding_capacity	Texture	Meat and carcass trait
107292	1	Mean_platelet_volume	Blood parameters	Health Traits
107291	1	Mean_platelet_volume	Blood parameters	Health Traits
107311	1	Platelet_count	Blood parameters	Health Traits
107303	1	Plateletcrit	Blood parameters	Health Traits
21348	1	PRRSV_susceptibility	Disease susceptibility	Health Traits
140357	1	PRRSV_susceptibility	Disease susceptibility	Health Traits
30985	1	Maternal_infanticide	Behavioral	Exterior traits
30984	1	Maternal_infanticide	Behavioral	Exterior traits
30983	1	Maternal_infanticide	Behavioral	Exterior traits
30982	1	Maternal_infanticide	Behavioral	Exterior traits

ID QTL	SSC	Trait	Class	Group
30986	1	Maternal_infanticide	Behavioral	Exterior traits
126114	1	Top_line_conformation	Conformation	Exterior traits
126113	1	Top_line_conformation	Conformation	Exterior traits
64697	1	Lumbar_vertebra_number	Conformation	Exterior traits
64698	1	Lumbar_vertebra_number	Conformation	Exterior traits
64627	1	Thoracic_vertebra_number	Conformation	Exterior traits
126125	1	Conformation_score	Conformation	Exterior traits
55895	1	Scrotal/inguinal_hernia	Defects	Exterior traits
22916	13	Total_number_born	Litter traits	Reproduction traits
18231	13	Total_number_born	Litter traits	Reproduction traits
18207	13	Total_number_born	Litter traits	Reproduction traits
18202	13	Total_number_born	Litter traits	Reproduction traits
18201	13	Total_number_born	Litter traits	Reproduction traits
18200	13	Total_number_born	Litter traits	Reproduction traits
18199	13	Total_number_born	Litter traits	Reproduction traits
18084	13	Total_number_born_alive	Litter traits	Reproduction traits
18082	13	Total_number_born_alive	Litter traits	Reproduction traits
18080	13	Total_number_born_alive	Litter traits	Reproduction traits
18079	13	Total_number_born_alive	Litter traits	Reproduction traits
18078	13	Total_number_born_alive	Litter traits	Reproduction traits
18077	13	Total_number_born_alive	Litter traits	Reproduction traits
18076	13	Total_number_born_alive	Litter traits	Reproduction traits
170930	13	Total_number_born_alive	Litter traits	Reproduction traits
170929	13	Total_number_born_alive	Litter traits	Reproduction traits
57525	13	Number_of_stillborn	Litter traits	Reproduction traits
57521	13	Number_of_stillborn	Litter traits	Reproduction traits
18134	13	Number_of_stillborn	Litter traits	Reproduction traits
18133	13	Number_of_stillborn	Litter traits	Reproduction traits
18314	13	mummified_pigs	Litter traits	Reproduction traits

ID QTL	SSC	Trait	Class	Group
18313	13	mummified_pigs	Litter traits	Reproduction traits
18312	13	mummified_pigs	Litter traits	Reproduction traits
18283	13	mummified_pigs	Litter traits	Reproduction traits
18262	13	mummified_pigs	Litter traits	Reproduction traits
18261	13	mummified_pigs	Litter traits	Reproduction traits
18260	13	mummified_pigs	Litter traits	Reproduction traits
18259	13	mummified_pigs	Litter traits	Reproduction traits
18258	13	mummified_pigs	Litter traits	Reproduction traits
106219	13	Litter_size	Litter traits	Reproduction traits
106227	13	Litter_size_at_day_5_after_birth	Litter traits	Reproduction traits
31888	13	Corpus_luteum_number	Litter traits	Reproduction traits
31885	13	Corpus_luteum_number	Litter traits	Reproduction traits
31884	13	Corpus_luteum_number	Litter traits	Reproduction traits
22927	13	Total_number_born_alive	Litter traits	Reproduction traits
18081	13	Total_number_born_alive	Litter traits	Reproduction traits
18205	13	Total_number_born	Litter traits	Reproduction traits
18204	13	Total_number_born	Litter traits	Reproduction traits
18198	13	Total_number_born	Litter traits	Reproduction traits
57529	13	Number_of_stillborn	Litter traits	Reproduction traits
18085	13	Total_number_born_alive	Litter traits	Reproduction traits
18208	13	Total_number_born	Litter traits	Reproduction traits
18203	13	Total_number_born	Litter traits	Reproduction traits
31887	13	Corpus_luteum_number	Litter traits	Reproduction traits
18177	13	Gestation_length	Reprodictive traits	Reproduction traits
126680	13	Teat_number	Reproductive organ	Reproduction traits
126650	13	Teat_number__difference_between_sides	Reproductive organ	Reproduction traits
126716	13	Left_teat_number	Reproductive organ	Reproduction traits
55859	13	Cryptorchidism	Reproductive organ	Reproduction traits
126606	13	Teat_number__difference_between_sides	Reproductive organ	Reproduction traits

ID QTL	SSC	Trait	Class	Group
135656	13	Time_in_feeder_per_day	Feed intake	Production traits
65130	13	Body_weight	Growth	Production traits
29602	13	Body_weight_birth	Growth	Production traits
23004	13	Body_weight_birth	Growth	Production traits
23003	13	Body_weight_birth	Growth	Production traits
28068	13	Residual_feed_intake	Feed intake	Production traits
28078	13	Residual_feed_intake	Feed intake	Production traits
28079	13	Residual_feed_intake	Feed intake	Production traits
21965	13	Firmness	Texture	Meat and carcass trait
55965	13	Drip_loss	Texture	Meat and carcass trait
32091	13	Palmitic_acid_content	Fatty acid content	Meat and carcass trait
32090	13	Palmitic_acid_content	Fatty acid content	Meat and carcass trait
106287	13	Oleic_acid_content	Fatty acid content	Meat and carcass trait
32127	13	Linoleic_acid_content	Fatty acid content	Meat and carcass trait
32126	13	Palmitic_acid_content	Fatty acid content	Meat and carcass trait
22458	13	Intramuscular_fat_content	Fatness	Meat and carcass trait
125483	13	Intramuscular_fat_content	Fatness	Meat and carcass trait
135764	13	Backfat_at_rump	Fatness	Meat and carcass trait
22291	13	Average_backfat_thickness	Fatness	Meat and carcass trait
193573	13	Average_backfat_thickness	Fatness	Meat and carcass trait
121782	13	Average_backfat_thickness	Fatness	Meat and carcass trait
121773	13	Average_backfat_thickness	Fatness	Meat and carcass trait
121772	13	Average_backfat_thickness	Fatness	Meat and carcass trait
121770	13	Average_backfat_thickness	Fatness	Meat and carcass trait
121769	13	Average_backfat_thickness	Fatness	Meat and carcass trait
24223	13	Intramuscular_fat_content	Fatness	Meat and carcass trait
135760	13	Intestinal_fat_weight	Fatness	Meat and carcass trait
135762	13	Backfat_at_rump	Fatness	Meat and carcass trait
135765	13	Backfat_at_last_rib	Fatness	Meat and carcass trait

ID QTL	SSC	Trait	Class	Group
18662	13	indole__laboratory	Chimestry	Meat and carcass trait
23173	13	Shoulder_weight	Anatomy	Meat and carcass trait
24222	13	Loin_percentage	Anatomy	Meat and carcass trait
29588	13	Loin_muscle_depth	Anatomy	Meat and carcass trait
29587	13	Loin_muscle_depth	Anatomy	Meat and carcass trait
29586	13	Loin_muscle_depth	Anatomy	Meat and carcass trait
95713	13	Ham_weight	Anatomy	Meat and carcass trait
29589	13	Loin_muscle_depth	Anatomy	Meat and carcass trait
140335	13	PRRSV_susceptibility	Disease susceptibility	Health Traits
107286	13	Mean_corpuscular_volume	Blood parameters	Health Traits
107263	13	Mean_corpuscular_hemoglobin_content	Blood parameters	Health Traits
56002	13	LDL_cholesterol	Blood parameters	Health Traits
55992	13	LDL_cholesterol	Blood parameters	Health Traits
107328	13	Red_cell_distribution_width	Blood parameters	Health Traits
107287	13	Mean_corpuscular_volume	Blood parameters	Health Traits
107253	13	Lymphocyte_number	Imune capacity	Health Traits
64768	13	Spinal_curvature	Defects	Exterior traits
64687	13	Spinal_curvature	Defects	Exterior traits
55898	13	Scrotal/inguinal_hernia	Defects	Exterior traits
31001	13	Maternal_infanticide	Behavioral	Exterior traits
126149	13	Conformation_score	Conformation	Exterior traits
31003	13	Maternal_infanticide	Behavioral	Exterior traits
18235	15	Total_number_born	Litter traits	Reproduction traits
18212	15	Total_number_born	Litter traits	Reproduction traits
22928	15	Total_number_born_alive	Litter traits	Reproduction traits
18093	15	Total_number_born_alive	Litter traits	Reproduction traits
18092	15	Total_number_born_alive	Litter traits	Reproduction traits
170932	15	Total_number_born_alive	Litter traits	Reproduction traits
57533	15	Number_of_stillborn	Litter traits	Reproduction traits

ID QTL	SSC	Trait	Class	Group
57532	15	Number_of_stillborn	Litter traits	Reproduction traits
57530	15	Number_of_stillborn	Litter traits	Reproduction traits
57522	15	Number_of_stillborn	Litter traits	Reproduction traits
57519	15	Number_of_stillborn	Litter traits	Reproduction traits
18135	15	Number_of_stillborn	Litter traits	Reproduction traits
18128	15	Number_of_stillborn	Litter traits	Reproduction traits
18317	15	mummified_pigs	Litter traits	Reproduction traits
18266	15	mummified_pigs	Litter traits	Reproduction traits
18264	15	mummified_pigs	Litter traits	Reproduction traits
22961	15	Litter_weight__total	Litter traits	Reproduction traits
22960	15	Litter_weight__total	Litter traits	Reproduction traits
31894	15	Corpus_luteum_number	Litter traits	Reproduction traits
31891	15	Corpus_luteum_number	Litter traits	Reproduction traits
170933	15	Total_number_born_alive	Litter traits	Reproduction traits
18111	15	Number_of_stillborn	Litter traits	Reproduction traits
31895	15	Corpus_luteum_number	Litter traits	Reproduction traits
57518	15	Number_of_stillborn	Litter traits	Reproduction traits
57531	15	Number_of_stillborn	Litter traits	Reproduction traits
18233	15	Total_number_born	Litter traits	Reproduction traits
22918	15	Total_number_born	Litter traits	Reproduction traits
22959	15	Litter_weight__total	Litter traits	Reproduction traits
18152	15	Gestation_length	Reprodictive traits	Reproduction traits
96083	15	Age_at_puberty	Reprodictive traits	Reproduction traits
160686	15	Sperm_abnormality_rate	Reproduction traits	Reproduction traits
37467	15	Teat_number	Reproductive organ	Reproduction traits
126593	15	Teat_number__difference_between_sides	Reproductive organ	Reproduction traits
126698	15	Left_teat_number	Reproductive organ	Reproduction traits
126685	15	Left_teat_number	Reproductive organ	Reproduction traits
126684	15	Teat_number__difference_between_sides	Reproductive organ	Reproduction traits

ID QTL	SSC	Trait	Class	Group
23016	15	Body_weight_birth	Growth	Production traits
23015	15	Body_weight_birth	Growth	Production traits
23014	15	Body_weight_birth	Growth	Production traits
23013	15	Body_weight_birth	Growth	Production traits
170898	15	Days_to_100_kg	Growth	Production traits
139170	15	Days_to_110_kg	Growth	Production traits
167229	15	Cooking_yield	Texture	Meat and carcass trait
167230	15	Drip_loss	Texture	Meat and carcass trait
167228	15	pH_24_hr_post-mortem_loin	pH	Meat and carcass trait
32130	15	P+44:93almitic_acid_content	Fatty acid content	Meat and carcass trait
32094	15	Myristic_acid_content	Fatty acid content	Meat and carcass trait
32131	15	Palmitic_acid_content	Fatty acid content	Meat and carcass trait
65140	15	Lean_meat_percentage	Fatness	Meat and carcass trait
29599	15	Intramuscular_fat_content	Fatness	Meat and carcass trait
147273	15	Intramuscular_fat_content	Fatness	Meat and carcass trait
125487	15	Intramuscular_fat_content	Fatness	Meat and carcass trait
65145	15	Fat_weight_total	Fatness	Meat and carcass trait
65142	15	Fat_percentage_in_carcass	Fatness	Meat and carcass trait
65138	15	Fat_percentage_in_carcass	Fatness	Meat and carcass trait
121913	15	Backfat_between_3rd_and_4th_last_ribs	Fatness	Meat and carcass trait
121911	15	Backfat_between_3rd_and_4th_last_ribs	Fatness	Meat and carcass trait
135764	15	Backfat_at_rump	Fatness	Meat and carcass trait
170629	15	Average_backfat_thickness	Fatness	Meat and carcass trait
65144	15	Fat_weight_total	Fatness	Meat and carcass trait
65141	15	Lean_meat_percentage	Fatness	Meat and carcass trait
121777	15	Average_backfat_thickness	Fatness	Meat and carcass trait
65143	15	Lean_meat_percentage	Fatness	Meat and carcass trait
65147	15	Fat_percentage_in_carcass	Fatness	Meat and carcass trait
65146	15	Lean_meat_percentage	Fatness	Meat and carcass trait

ID QTL	SSC	Trait	Class	Group
170646	15	Lean_meat_percentage	Fatness	Meat and carcass trait
147274	15	Intramuscular_fat_content	Fatness	Meat and carcass trait
65139	15	Fat_weight_total	Fatness	Meat and carcass trait
135765	15	Backfat_at_last_rib	Fatness	Meat and carcass trait
147278	15	Intramuscular_fat_content	Fatness	Meat and carcass trait
147283	15	Intramuscular_fat_content	Fatness	Meat and carcass trait
147284	15	Intramuscular_fat_content	Fatness	Meat and carcass trait
147285	15	Intramuscular_fat_content	Fatness	Meat and carcass trait
147286	15	Intramuscular_fat_content	Fatness	Meat and carcass trait
147287	15	Intramuscular_fat_content	Fatness	Meat and carcass trait
147288	15	Intramuscular_fat_content	Fatness	Meat and carcass trait
147289	15	Intramuscular_fat_content	Fatness	Meat and carcass trait
147290	15	Intramuscular_fat_content	Fatness	Meat and carcass trait
147291	15	Intramuscular_fat_content	Fatness	Meat and carcass trait
147292	15	Intramuscular_fat_content	Fatness	Meat and carcass trait
147293	15	Intramuscular_fat_content	Fatness	Meat and carcass trait
147282	15	Intramuscular_fat_content	Fatness	Meat and carcass trait
147281	15	Intramuscular_fat_content	Fatness	Meat and carcass trait
147280	15	Intramuscular_fat_content	Fatness	Meat and carcass trait
147279	15	Intramuscular_fat_content	Fatness	Meat and carcass trait
135761	15	Backfat_at_first_rib	Fatness	Meat and carcass trait
135763	15	Backfat_at_first_rib	Fatness	Meat and carcass trait
135760	15	Intestinal_fat_weight	Fatness	Meat and carcass trait
135762	15	Backfat_at_rump	Fatness	Meat and carcass trait
135759	15	Shoulder_subcutaneous_fat_thickness	Fatness	Meat and carcass trait
147275	15	Intramuscular_fat_content	Fatness	Meat and carcass trait
147277	15	Intramuscular_fat_content	Fatness	Meat and carcass trait
147276	15	Intramuscular_fat_content	Fatness	Meat and carcass trait
167231	15	muscle_protein_percentage	Chimestry	Meat and carcass trait

ID QTL	SSC	Trait	Class	Group
18662	15	indole__laboratory	Chimestry	Meat and carcass trait
107331	15	Red_cell_distribution_width	Blood parameters	Health Traits
140329	15	PRRS_viral_load	Disease susceptibility	Health Traits
15128	15	Bilirubin_level	Blood parameters	Health Traits
171144	15	Blood_urea_level	Blood parameters	Health Traits
64781	15	Thoracolumbar_vertebra_number	Conformation	Exterior traits
30994	15	Maternal_infanticide	Behavioral	Exterior traits
64663	15	Lumbar_vertebra_number	Conformation	Exterior traits
